# Contextual support for children’s recall within working memory

**DOI:** 10.1177/1747021818804440

**Published:** 2018-10-24

**Authors:** Hannah E Roome, John N Towse, Maria M Crespo-Llado

**Affiliations:** 1 Department of Psychology, Lancaster University, Lancaster, UK; 2 Center for Learning and Memory, Department of Neuroscience, The University of Texas at Austin, Austin, TX, USA

**Keywords:** Working memory capacity, children, recall reconstruction, secondary memory

## Abstract

Measures of working memory capacity (WMC) are extremely popular, yet we know relatively little about the specific processes that support recall. We focused on children’s and adults’ ability to use contextual support to access working memory representations that might otherwise not be reported. Children (*N* = 186, 5-10 years) and adults (*N* = 64) completed a listening span task and a delayed recall task with semantic probes or cues. Clear age-related increases in listening span were evident. All age groups benefitted from contextual support to retrieve degraded target memoranda, particularly on listening span tasks when the cues provided semantic support for processing events, in comparison to cues associated specifically with memoranda. Response latencies suggested a developing efficiency in children’s use of contextual support for delayed recall correlated with listening span performance. These probe tasks support accounts of working memory that recognise reconstructive and cued search processes.

## Introduction

Working memory is a highly influential construct that has been instrumental for understanding adult cognition and children’s cognitive development (Alloway, Gathercole, & Pickering, 2006; Hitch et al., 1983). It is widely recognised that working memory representations are fragile, and loss of fidelity can result from several different processes. One historically powerful way to conceptualise this is through the “acid bath” model of forgetting (Posner & Konick, 1966), whereby cognitive activity disrupts or dissolves the original trace to the point that it can no longer be identified. This type of perspective encourages the contrast between representations that are available (remembered) or inaccessible (forgotten). Yet, is this simple distinction sufficient?

In our view, the distinction between working memory representations that are remembered or forgotten has been critical to research progress. To draw an analogy within a daily cycle, the distinction between night and day is self-evident and dramatic. But of course, there is also twilight, a phase that is neither completely one nor the other. We describe data that illustrate, likewise, how children and adults can both fail to recall targets spontaneously yet be prompted to produce that information. Memoranda may not be remembered using traditional methods, yet they have not been entirely forgotten. We argue that this complements contemporary accounts of working memory recall that specify how fragile (i.e., incomplete or uncertain) working memory representations can be reconstructed and scaffolded.

Unsworth and Engle (2007) proposed that individual differences in working memory capacity (WMC) involve more than just active maintenance. They distinguished primary memory (PM)—the attentionally demanding active maintenance of a small number of representations—and secondary memory (SM), the effortful, cue-dependent, contextual search process required to retrieve displaced memoranda. Roome, Towse, and Jarrold (2014) calculated recall lags in free recall to illustrate developmental changes in PM and SM. Recall lags indicate the item distances between presentation and subsequent production of a to-be-recalled (TBR) item (see Tulving & Colotla, 1970 for the original method, also Jarrold et al., 2015 for a critical analysis). Children aged 5-8 years predominantly relied on PM and showed very limited evidence of SM. Despite this, SM measures correlated with WMC, consistent with the view that contextual search contributes to WMC. Yet, direct developmental evidence on the nature or potency of potential cued search processes is limited.

Complementary to the notion of SM, the recall reconstruction hypothesis argues that working memory representations are not always held in a complete and encapsulated form, but they may be recovered or enhanced by additional information (Cowan et al., 2003; Towse, Cowan, Hitch, & Horton, 2008). This hypothesis predicts that appropriate cues should be able to clarify, specify, and disambiguate TBR items within working memory (see also Towse, Hitch, Horton, & Harvey, 2010). Thus, reconstructive processes can use context to facilitate the recovery of memory items that were in immediate memory. This was tested by presenting adults (Towse et al., 2008) and children (Towse et al., 2010) different versions of a reading span task, a traditional and frequently used measure of WMC. An “integrated” condition involved TBR items drawn from the processing event, while an “independent” condition used TBR items unconnected to processing. Both adults and children used the sentence context to facilitate retrieval of TBR items. Recall was more accurate in the integrated condition than the independent condition, despite longer pauses between the production of each word—with pauses thought to reflect reconstructive activity. Thus, processing and retention elements of complex span tasks are not always independent and in competition with each other, as proposed in some accounts (e.g., resource-sharing hypothesis: Case, Kurland, & Goldberg, 1982; Daneman & Carpenter, 1980; task-switching: Towse, Hitch, & Hutton, 1998). Indeed, the integration of processing and retention elements of the task allows a rich environment whereby semantic representations within processing can disambiguate TBR items that may otherwise have been forgotten (Osaka, Nishizaki, Komori, & Osaka, 2002; Tanaka, Saito, & Kikuchi, 2014).

To provide more direct measures of children’s use of contextual support to supplement WMC, we investigated two tasks: a cued listening span task and delayed cued recall task. Both tasks use external cues to facilitate the reinstatement of a target memory trace. For a sentence completion, cued listening span task, participants generated a word to finish a set of incomplete sentences, which was followed by the recall of the self-generated words in serial order. Up to this point, it has the format of a traditional complex span task. However, a third, cued phase then took place to assess the recoverability of memoranda that was not initially recalled. Second, the delayed cued recall task explored developmental changes in the efficiency in which children and adults can use the external semantic support to recover the target item. The delay within the task forced participants to retrieve memoranda that can no longer be actively maintained, and therefore, the displaced representation must be selected using the contextual support.

Complex span tasks interleave a series of processing and encoding/memory episodes. Early models of WMC stressed the simultaneous demand of active maintenance and concurrent processing (Case et al., 1982; Daneman & Carpenter, 1980). Subsequent analyses argue for switching between memory and retention (Towse & Hitch, 1996; Towse, Hitch, & Hutton, 1998) and/or emphasise a sequential cycle of memory refreshment and degradation (Barrouillet & Camos, 2015; Loaiza & Camos, 2018). In addition, Unsworth and Engle (2006, 2007) argue that processing new information prevents active maintenance in PM. The displaced memoranda are then retrieved through SM. We therefore ask under this model—what faciliates item recovery or supports the retrieval of memory traces that are not immediately accessible?

Taking the structure of the complex span task and the processes it measures within the working memory system, we manipulated external cue support to examine the extent to which different types of context facilitate SM retrieval in adults and children. Three different contextual cue conditions were incorporated into the traditional task; a “sentence-cue” provided items that were reminders of words drawn from (earlier) in the sentence, while a “word-cue” involved items related to (i.e., associates of) the memorandum. For example, when presented with the sentence, “Before school, I eat my . . .” the TBR word expected for self-generated completion is “breakfast.” A sentence-cue would be “school,” while the word-cue would be “cereal.” The third “no-cue” condition acted as a control group to provide a baseline WMC for each age group to compare against any increased recall performance facilitated by contextual support.

We assume that successful sentence-cue benefits are mediated by memory of the sentence gist because on its own, “school” is unlikely to prompt the production of “breakfast.” Therefore, sentence-cues are predicted to be effective only to the extent that they facilitate memory of the sentence, which in turn helps reconstruct the TBR memoranda (Towse et al., 2008; Towse et al., 2010). That is, the recall cue acts through sentence memory and is indirect. In contrast, the word-cue was designed to be an associate of the TBR word—though we deliberately avoided the highest association cues to reduce the possibility this became a word-association paradigm. Importantly, the word-cue is specific to the TBR item and not linked to the sentence. Word-cue benefits therefore suggest that identification of the TBR item can be recovered and that it may exist in some fragile, but non-reportable, state without the cue.

The second, complementary, paradigm was a delayed cued recall task, inspired by the adult-based work of Miyake and Friedman (2004). They presented adults with three words, quickly followed by a simple unrelated distractor activity for a varying length of time. At recall, participants were given an external superordinate category cue to elicit just one of the three presented items. Miyake and Friedman (2004) found that this cued recall task was the best mediator of the relationship between reading span and cognitive abilities among an ensemble of cognitive (e.g., word span, sentence verification times, proactive interference, word knowledge) and psychometric (e.g., reading comprehension, non-verbal reasoning) variables. They argued that semantically controlled search (as captured by the cued recall task) is a feature of adults’ reading span performance. This was the basis for our child-appropriate version of the task, providing a developmental perspective of children’s controlled search on this task and its relation to traditional measures of WMC.

To ensure its developmental application, we used stimuli appropriate for children, plus semantic relationships of which children are aware of. The use of a 15 s delay prevented participants from actively maintaining the presented memoranda in PM. Free recall studies confirm the restriction on children’s SM (i.e., the primacy effect)—children up to the age of 10 years typically retrieve a single SM item (Roome, 2016). Therefore, the presentation of a smaller number of items, with the addition of external semantic support to facilitate the retrieval of one SM trace, provides an appropriate measure of children’s ability to combine external as well as any internally generated contextual information (Unsworth, 2009).

We also measured the time taken for successful retrieval. Cowan et al. (2003) argued that inter-word pauses in complex span reflect memory search and retrieval operations. Indeed, Towse et al. (2010) found longer pauses between words in the integrated reading span condition in comparison to the independent condition. Thus, the interval between cue presentation and participants’ responses, referred to as a preparatory interval, may offer a useful metric for examining developmental change in the accessibility of potentially degraded memoranda as a consequence of being outside immediate memory (Towse et al., 2008; Towse et al., 2010).

In summary, we investigate whether children (5-10 years) and adults use contextual cues to support working memory recall. A 5-year age range across primary school in the United Kingdom enabled us to track any developmental change. The empirical work addresses four key issues. First, Experiment 1A used both tasks to experimentally test whether contextual support can facilitate the retrieval of memory representations that are no longer in immediate focus. The cued listening span task will show whether memory traces that children do not report spontaneously can be elicited by different types of contextual information. Based on the recall reconstruction hypothesis, we predict that the sentence representations potentially also remain accessible during the recall phase. Therefore, the recall reconstruction hypothesis argues that the sentence-cue will help memorandum recall by improving access to the contextual environment (the sentence that generated it). In comparison, the word-cue support is more specifically linked to the memorandum itself. Second, the delayed cued recall accuracy and preparatory interval will characterise children’s recall efficiency. Third, Experiment 1B will provide children with a second opportunity to spontaneously retrieve forgotten memoranda. This follow-up condition will confirm whether it is cues, or just the additional recall opportunities, that affect recall. Fourth, individual differences will be considered to examine task relationships between estimates of SM and WMC. Overall, our analysis should show the extent to which age–WMC associations can be accounted for by novel SM estimates to allow us to address a new developmental account of the variables supporting WMC.

## Experiment 1A

### Methods

We report how we determined our sample size, all data exclusions, measures, and manipulations in the study (Simmons, Nelson, & Simonsohn, 2012).

#### Participants

Participants comprised 142 children, from three schools in the North West of England, UK, and 48 adults from Lancaster University, Lancaster, UK (*N* = 48, *M* = 19.03 years, range: 18.06-20.08 years, 35 females). Children were stratified by school class into three age groups: 48 children aged 5-6 years (Year 1, *M* = 6.00 years, range: 5.05-6.07 years, 27 females), 43 children aged 7-8 years (Year 3, *M* = 7.11 years, range: 7.02-8.06 years, 21 females), and 51 children aged 9-10 years (Year 5, *M* = 10.00 years, range: 9.02-10.07 years, 34 females). Participants, or their parents, provided written consent to take part. We recruited as many children as possible from eligible classes and subsequent adult recruitment designed to form a comparable sample size. The adult data formed part of a larger research project (Roome, 2016). All participants included in this data collection spoke English as their first language and did not report any learning difficulties. In all, 10 participants from Year 5 children were not included in the delayed cued recall task analyses due to incomplete testing sessions.

#### Materials

We utilised a corpus of 139 TBR words: 88 for the cued listening span task (Supplementary Material 1) and 51 for the delayed cued recall task (Supplementary Material 2). The cued listening span sentences were taken from Towse, Hutton, and Hitch (1998) and recorded in a female voice. The mean number of words in each incomplete sentence was 5.41 words, with a mean recording length of 1.71 s (Supplementary Material 1), spoken at 3.09 words per second. The *sentence-cue* prompts were selected from the main semantic focus of sentences. The *word-cue* prompts were selected from the Edinburgh Associative Thesaurus. The external cues were selected by assessing whether they had a semantic relation with the target storage items but were not related to the cue chosen in the sentence-cue condition.

Delayed cued recall stimuli were organised by semantic category, based on Van Overschelde, Rawson, and Dunlosky (2004). Pilot data established common word associations; 40 adult participants wrote down the first three concrete nouns that came to mind from 20 target categories. Only items specified by less than 50% of participants were used as candidate stimuli. For example, for semantic category *fruit*, “apple” and “banana” were excluded, as they were produced 85% and 55% of the time, respectively. Instead, “cherry,” “grapes,” and “pear” were included for this specific semantic category. Based on age norms (Morrison, Chappell, & Ellis, 1997), 15 semantic categories and their associated concrete nouns were selected (Supplementary Material 2).

### Design and procedure

Both tasks were programmed using PsychoPy (Pierce, 2007, 2009).^1^ The cued listening span task followed a between-participants design—participants were split into *sentence-cue, word-cue*, or *no-cue* conditions. Children were tested on the tasks in a single session, counterbalanced accordingly. Individual testing occurred in a quiet classroom setting. Adults were also tested individually in a laboratory setting.

#### Cued listening span task

**Figure 1.**
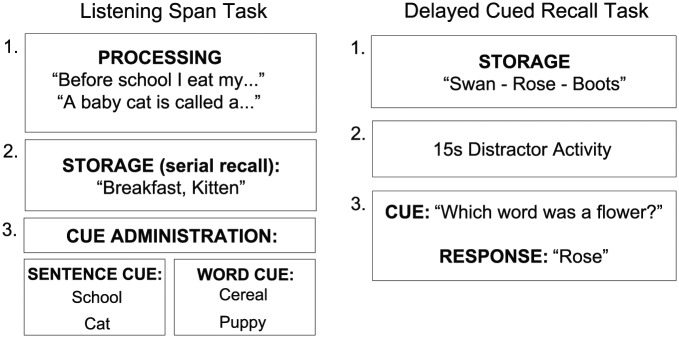
A schematic demonstration of the cued listening span task and the delayed cued recall task.

Participants heard a sentence and generated a semantically appropriate final word. Once participants had completed all the sentences within a trial, they were required to recall their self-generated words in serial order (Figure 1). Within a trial, the task started with two sentences/TBR words, increasing incrementally up to five sentences/TBR words, creating four levels of difficulty. Participants had to complete three trials of each sentence/word length, which generated 12 trials overall, split into four blocks of testing. The experimenter recorded the number of correct items recalled and their serial position for each trial. The proportion of correct baseline recall was calculated for each participant by calculating the sum of correctly recalled items, in the correct serial position, divided by the total number of TBR items within the task. Recall of the correct item in the wrong serial position was marked as incorrect.

Table 1 shows the breakdown of participants into the three different cue configurations, to help aid the recall of target items that were not reported at the first point of recall within the task. The cues for unrecalled items were spoken by the experimenter in the original order of administration (i.e., the first TBR item forgotten was the first item to be cued). Initially, participants were not aware that they were going to be given cues. However, this became apparent at the end of a testing block when it was explained that they would be given cues. Participants in the sentence-cue condition listened to prompts from the original sentences, while participants in the word-cue condition listened to prompts of target word associates that had not been used in the sentence. In the no-cue condition, participants carried out the task as a conventional listening span task with no aid. The experimenter recorded the number of cues administered and TBR items participants retrieved correctly following cue administration.

**Table 1.** A breakdown of participants for the cued listening span task.

	Cue conditions		
	No-cue	Sentence-cue	Word-cue
Year 1	16	16	16
(5- to 6-year-olds)			
Year 3	15	14	14
(7- to 8-year-olds)			
Year 5	18	17	16
(9- to 10-year-olds)			
Adults	16	16	16
(18- to 29-year-olds)			

#### Delayed cued recall task

Initially, the experimenter read a list of the different semantic categories and asked whether they knew the categories involved and gave an example. If not, they were provided a description and given an example of that category to minimise the possibility that participants failed to recall a target because they did not know the semantic category. Figure 1 provides an outline of the task design on a trial-by-trial basis. Participants heard three unrelated, concrete nouns, each presented for 1 s, with a 250 ms interstimulus interval. After the stimuli presentation, a 15-s distractor activity was implemented. Adults counted backwards aloud in threes from a random three-digit number shown on the computer screen. Children counted aloud target objects hidden in a visual scene.^2^ Following the distractor activity, participants were cued by a semantic category to recall one target item. The experimenter pressed a button as soon as the participant started to produce their answer. This eliminated motor movement/button pressing as a chronometric variable. Participants completed 15 trials, organised into three testing blocks (five trials per block). The serial position of the target memory item was pseudo-randomised across trials, generating a total of five trials for each serial position. The number of correct responses and the total proportion of correct recall were recorded. The total proportion of correct recall was calculated by the total number of correctly recalled items divided by the total number of TBR items. The response time was the gap between the end of the cue administration and the beginning of the participants’ response.

### Results

All the underlying data in this article (Experiments 1A and 1B) are available from http://dx.doi.org/10.17635/lancaster/researchdata/230

#### Cued listening span task

##### Baseline performance and total capacity after cue administration

**Figure 2.**
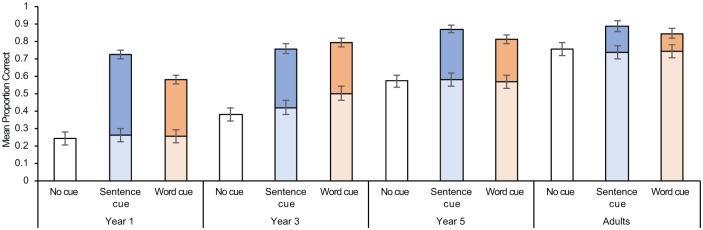
A cumulative bar chart demonstrating participants’ baseline proportion of correct recall (lower column) and the increased proportion of correct recall after cue administration (upper column). Error bars represent one standard error from the mean.

First, we report participants’ baseline performance—before cue administration—as shown in the lower columns of Figure 2. A 3 (Condition: no-cue vs. sentence-cue vs. word-cue) × 4 (Age: Year 1 vs. Year 3 vs. Year 5 vs. Adults) analysis of variance (ANOVA) with proportion of baseline correct recall as the dependent variable revealed an age-related increase in complex span, *F*(3, 178) = 85.188, mean square error (MSE) = .025, *p* < .001, $\eta_{p}^{2}=.589$, with significant differences between each age group (all *p*s < .001).^3^ We did not find a cue effect, *F* < 1, *MSE* = .025, *p* = .624, $\eta_{p}^{2}=.005$ (no-cue condition: *M* = .487, *SE* = .020; sentence-cue condition: *M* = .499, *SE* = .020; word-cue condition: *M* = .514, *SE* = .020), nor a significant interaction between the two factors, *F* < 1, *MSE* = .025, *p* = .678, $\eta_{p}^{2}=.022$. As one would expect, baseline performance varied with age but not the cue condition which was yet to be affected.

The upper elements of the stacked columns in Figure 2 displays the additional items reported after providing the sentence- and word-cues. We re-ran the previous ANOVA with combined recall as the dependent variable—pooling recall before and after cue administration. This re-confirmed the developmental increase in recall, *F*(3, 178) = 44.500, *MSE* = .020, *p* < .001, $\eta_{p}^{2}=.429$, but now showed significant differences between cue conditions, *F*(2, 178) = 94.788, *MSE* = .020, *p* < .001, $\eta_{p}^{2}=.516$. Both of the cued conditions facilitated an equivalently significant higher level of recall (sentence-cue: *M* = .807, *SE* = .018; word-cue condition: *M* = .757, *SE* = .018) when compared to the no-cue condition (*M* = .487, *SE* = .018, both *p*s < .01).

A large interaction between age and condition, *F*(6, 178) = 6.215, *MSE* = .020, *p* < .001, $\eta_{p}^{2}=.173$, showed that cue effects were not the same in all age groups. Year 1 children showed a cue benefit, *F*(2, 45) = 47.058, *MSE* = .021, *p* < .001, $\eta_{p}^{2}=.677$, with the lowest recall reported in the no-cue condition, followed by the word-cue condition, leaving the sentence-cue condition with the largest proportion of correct recall (all *p*s < .05). Years 3 and 5 analyses did not find differences between the cued conditions, but their recall was higher in comparison to the control condition, Year 3: *F*(2, 40) = 34.930, *MSE* = .022, *p* < .001, $\eta_{p}^{2}=.636$; Year 5: *F*(2, 48) = 24.579, *MSE* *=* .017, *p* < .001, $\eta_{p}^{2}=.50$. For adults, the significant cue effect, *F*(2, 45) = 3.41, *MSE* = .020, *p* = .042, $\eta_{p}^{2}=.132$, was attributed to advantage in the sentence versus no-cue conditions (*p* = .042). The total capacity of the word condition did not differ from either the no-cue (*p* = .259) or sentence-cue condition (*p* = 1.000).

##### The benefit of external contextual support in recall

**Figure 3.**
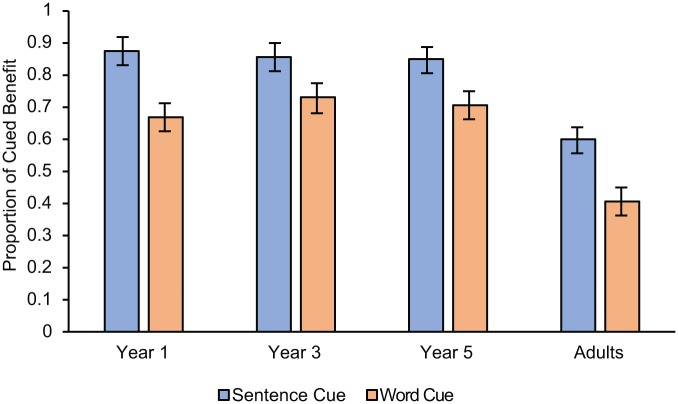
The proportion of cued benefit after cue administration as a function of age and condition. Error bars represent one standard error from the mean.

To understand the extent to which the cues were effective in boosting the recall of forgotten memoranda, we calculated the “cued benefit,” that is, the proportion of cues that successfully triggered a TBR item.^4^ Figure 3 shows the success of each recall prompt for all age groups. We then ran a 4 (Age: Year 1 vs. Year 3 vs. Year 5 vs. Adults) × 2 (Cue condition: sentence vs. word) ANOVA. Sentence-cues were more effective than word-cues, *F*(1, 117) = 15.785, *MSE* = .041, *p* < .001, $\eta_{p}^{2}=.119$ (*M* = .783, *SE* = .025; *M* = .639, *SE* = .026 respectively). We also found age differences, *F*(3, 117) = 15.695, *MSE* = .041, *p* = .017, $\eta_{p}^{2}=.287$. Although children benefitted similarly (Year 1: *M* = .767, *SE* = .036; Year 3: *M* = .807, *SE* = .038; Year 5: *M* = .770, *SE* = .035; all *p*s > .05), adults exhibited significantly less benefit (*M* = .499, *SE* = .036, all *p*s < .001). The interaction between the two factors was non-significant, *F* < 1, *MSE* = .041, *p* = .394, $\eta_{p}^{2}=.025$.

#### Delayed cued recall task

Table 2 describes participant accuracy and response latencies at delayed cued recall. ANOVAs revealed an age-related increase in accuracy, *F*(3, 176) = 39.848, *MSE* = .019, *p* < .001, $\eta_{p}^{2}=.404$, with significant increases at all four age points (all *p*s < .05), while the preparatory interval became systematically shorter with increasing age, *F*(3, 176) = 75.264, *MSE* = 3.070, *p* < .001, $\eta_{p}^{2}=.562$, with significant differences between each age group (all *p*s < .05).

**Table 2.** Age-related differences in the proportion of correct recall and mean preparatory interval (RT) from Experiments 1A and 1B.

	Year 1	Year 3	Year 5	Adults
Experiment 1A				
*N*	48	43	41	48
Mean proportion correct	.354 (.020)	.442 (.021)	.529 (.021)	.651 (.020)
Mean correct preparatory interval (s)	6.734 (.236)	4.171 (.249)	2.891 (.256)	1.559 (.236)
Experiment 1B				
*N*	12	18	11	16
Mean proportion correct	.256 (.038)	.457 (.031)	.515 (.041)	.763 (.034)
Mean correct preparatory interval (s)	2.928 (.472)	2.834 (.386)	3.268 (.493)	1.949 (.409)

RT: reaction time.

### Discussion

We sought to establish whether children and adults could capitalise on cues in a complex span task to recall items that they spontaneously failed to report. The fact that they did so supports the view that memoranda may reside in a fragile state: inaccessible but not yet forgotten through a typical immediate memory paradigm. Delayed cued recall performance emphasised that children struggled to use superordinate cues, either because of cue potency or the state of their memory representations. Nonetheless, when given external contextual support in the cued listening span task, children’s performance was comparable to adults. This suggests that providing 5- to 10-year-old children with an external recall prompt of a semantically rich episode can be highly effective, yet they cannot easily structure output using superordinate prompts.

The cued listening span task was adapted from widely deployed complex span tasks that have been interpreted as tapping the retrieval of SM representations (Unsworth & Engle, 2007). Children’s baseline complex span increased with age, and 5- to 6-year-olds correctly reported 25% of the target memoranda, which increased to 57% by 10 years-old. The striking finding was the extent to which cues from the processing events as well as the TBR item facilitated the retrieval of “forgotten” memoranda. Cues allowed the reactivation of degraded target memoranda to substantially increase span performance, compared to the control no-cue condition. Even more striking, cued span of 7- to 8-year-olds was comparable to that of adults. Thus, the data suggest that limits in the internal generation and mediation of contextual search constrain children’s WMC.

We also established that children and adults recalled more items in the sentence-cue than the word-cue condition. The sentence-cue thus provided an especially beneficial “contextual gist” of the processing (Towse et al., 2008; Towse et al., 2010), strong enough to focus the search set (the cue was not associated with other memory representations within the search set; Schroeder, Copeland, & Bies-hernandez, 2012), and without generating too many representations for sampling. The word-cue condition clearly enhanced access to TBR memoranda too, evident in Figures 2 and 3. However, these cues did not reactivate degraded representations to the same extent as the sentence-cues. Even though we assume word-cues are more direct than sentence-cues, they were less powerful as triggers for the memoranda. Evidence already suggests that the fidelity of memory representations may be improved by the processing event that generated them (Cowan et al., 2003; Osaka et al., 2002; Tanaka et al., 2014; Towse et al., 2008; Towse et al., 2010). The sentence-cue benefit further underlines the conceptual argument that the processing and memory elements of a complex span task need not always or entirely be competitive with one another (as suggested by, for example, Case et al., 1982; Daneman & Carpenter, 1980; Towse, Hitch, & Hutton, 1998).

Why didn’t adults show a comparable reconstructive benefit? After cue administration, adult recall in the word-cue condition did not differ from those in the no-cue baseline condition, although the sentence-cue condition was a more effective source of support. Adult baseline performance ranged from .735 to .756, which increased to .844 (word-cue condition) and .884 (sentence-cue condition), still giving them the opportunity to reactivate long-term memoranda, and therefore, a ceiling effect is not a plausible explanation. However, it is possible that the adults reached their peak performance on this specific complex span task, spontaneously utilising reconstructive processes to access fragile representations. Therefore, any residual TBR items were perhaps unrecoverable.

The delayed cued recall task used superordinate cues to show children’s efficiency in accessing target memory representations after a period of distractor activity designed to block active maintenance of memoranda. Younger children’s performance reflected the difficulty in using a cue to select one of three presented items. Moreover, response latencies were very long, indicating effortful and extensive search (Cowan et al., 2003; Towse et al., 2010). The low levels of performance reported converge with children’s low SM estimates in 5- to 8-year-olds (Roome et al., 2014). Thus, it is possible that an additional source of difficulty for children is in the *selectivity* of controlled search. Younger children organise items according to their associative relations, as opposed to their categorical structure (e.g., Bjorklund & de Marchena, 1984). Since the delayed cued recall task provides a categorical—superordinate—cue to the TBR candidates, children may have a mediational difficulty—the cue may not have been an effective trigger to the target item.

## Experiment 1B

It is important to determine whether the external contextual support in the first experiment facilitated the recovery and retrieval of memory representations, or whether the participants themselves could spontaneously retrieve the “forgotten” representations at the end of a block. Experiment 1A indicates that it can take a child up to 6 s to retrieve one memory representation that is not actively maintained. Therefore, it is possible that children did not have the time or attentional resources to retrieve multiple memoranda at the point of recall. But that does not mean it is truly forgotten. However, if the regeneration recall gain does not equate to the cued conditions in Experiment 1A, it provides further confirmation that cue-driven controlled search for information out of immediate focus follows a protracted development throughout childhood.^5^

To test this, we ran an additional control condition to the cued listening span task. Instead of participants being given cues to help facilitate retrieval, the participants were simply asked “*Are there any words that you thought you had forgotten, which you can now remember?*” This provided the participant with the opportunity to retrieve any memory representations that they had not initially recalled during their baseline recall, showing their ability to spontaneously regenerate secondary memoranda. Participants also completed the delayed cued recall task, keeping the experimental session as similar as possible to the previous experiment.

### Method

#### Participants

Data collection was carried out in one of the schools participating in Experiment 1A. We tested 46 children, 13 from Year 1 (*M* = 6.00 years, range: 5.08-6.06 years, 5 females), 22 from Year 3 (*M* = 8.02 years, range: 7.08-8.07 years, 10 females) and 11 from Year 5 (*M* = 10.02 years; range: 9.05-10.09 years; 8 females). The parents or guardians of all children provided written consent before they participated. An additional adult data set included 17 adults from Lancaster University (*M* = 22.01 years, range: 18.06-29.07 years; 14 females). Two children from Year 3 (*N* = 20) and one adult (*N* = 16) were excluded from the analyses as English was not their first language. No participants had any identified learning difficulties.

#### Materials and design

We deployed the same experimental setup as Experiment 1A—the same stimuli, task design, and programming software. Within a single testing session, all participants completed the new cued listening span condition: baseline listening span performance with regeneration opportunities and the delayed cued recall task. The order of the tasks administration was counterbalanced accordingly.

#### Procedure

The equipment and tasks were the same as Experiment 1A. Participants were tested on a one-to-one basis in either a quiet school classroom or a laboratory setting.

##### Cued listening span task

On experimental trials, participants listened to sentences and had to complete the sentence by generating a semantically relevant word. Once participants completed the sentences, they had to recall the self-generated words in serial order. The experimenter recorded the number of correct items recalled and their serial position for each trial, maintaining the same procedure to generate each participant’s baseline WMC. At the end of a block of trials, a message appeared on the screen that the experimenter read to the participant, “*Well done for remembering as many words as you can. Are there any words that you thought you had forgotten, which you can now remember?*” Following this question, participants had a second opportunity to verbally recall any words they did not recall previously. Once the participants had completed this second recall phase, they moved onto the next block.

##### Delayed cued recall task

Task administration was the same as Experiment 1A.

### Results

#### Cued listening span task

##### Baseline performance and total capacity after cue administration

**Figure 4.**
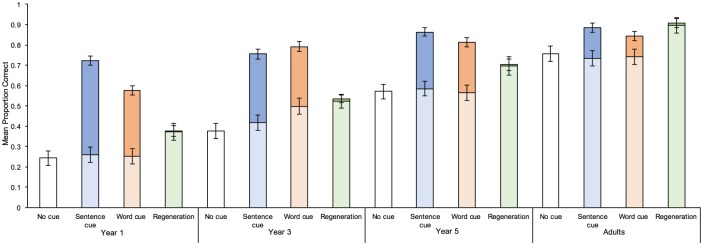
A cumulative bar chart demonstrating participants’ baseline proportion of correct recall (lower column) and the increased proportion of correct recall after cue administration (upper column) from Experiments 1A and 1B. Error bars represent one standard error from the mean.

Participants’ baseline performance in the current experiment was analysed and compared to the data collected in Experiment 1A in a 4 (Age group: Year 1 vs. Year 3 vs. Year 5 vs. Adults) × 4 (Condition: no-cue vs. sentence-cue vs. word-cue vs. regeneration) ANOVA (Figure 4). As expected, we obtained an age-related increase in complex span, *F*(3, 234) = 130.599, *MSE* = .022, *p* < .001, $\eta_{p}^{2}=.625$. We also found that participants in the regeneration condition exhibited a larger baseline WMC than the comparison conditions from Experiment 1A, *F*(3, 235) = 10.524, *MSE* = .022, *p* < .001, $\eta_{p}^{2}=.119$ (all *p*s < .001). The interaction between cue condition and age was non-significant, *F* < 1, *MSE* = .022, *p* = .773, $\eta_{p}^{2}=.024$, suggesting the condition differences were stable across all age groups.

Participants’ total capacity after cue administration differed among four different conditions, *F*(3, 234) = 71.83, *MSE* = .018, *p* < .001, $\eta_{p}^{2}=.479$ (no-cue: *M* = .487, *SE* = .017; regeneration: *M* = .630, *SE* = .018; word-cue: *M* = .757; *SE* = .017; sentence-cue: *M* = .807; *SE* = .017). A significant age and condition interaction, *F*(3, 234) = 6.587, *MSE* = .018, *p* < .001, $\eta_{p}^{2}=.202$, was the same in Experiment 1A, but with the regeneration condition consistently generating a lower total capacity (Figure 4).

**Figure 5.**
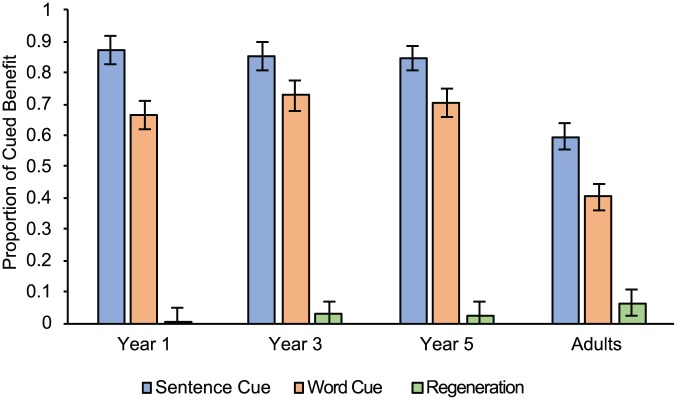
The proportion of cued benefit after cue administration in the sentence- and word-cue conditions from Experiment 1A and the regeneration condition from Experiment 1B. Error bars represent one standard error from the mean.

To gauge the benefit of giving participants a second recall opportunity, the regeneration benefit was calculated in a similar manner as the cued benefit in Experiment 1A: the total number of successfully regenerated TBR items divided by the total number of items that were not recalled in the first instance and therefore available to be retrieved when given the second opportunity. The analysis of the regeneration condition in isolation revealed no effect of age, *F*(3, 56) = 1.893, *MSE* = .005, *p* = .141, $\eta_{p}^{2}=.092$. The regeneration condition was then analysed in conjunction with the cued conditions in Experiment 1A (Figure 5). The effect of age remained similar to the report in Experiment 1A, *F*(3, 173) = 12.431, *MSE* = .029, *p* < .001, $\eta_{p}^{2}=.177$. The effect of condition, *F*(2, 174) = 324.543, *MSE* = .029, *p* < .001, $\eta_{p}^{2}=.790$, confirmed the regeneration condition led to the fewest additional items in comparison to the cued conditions (all *p*s < .001).

#### Delayed cued recall task

Accuracy and reaction time (RT) data (see Table 2) were analysed separately in two ANOVAs that used age and data set as the between-participant factors. Participants’ overall accuracy at the delayed cued recall task did not differ across experiments, *F* < 1, *MSE* = .019, *p* = .859, $\eta_{p}^{2}<.001$. The significant effect of age, *F*(3, 232) = 66.425, *MSE* = .019, *p* < .001, *$\eta_{p}^{2}=.462$*, revealed systematic increases in performance across all age groups (all *p*s < .05). However, the interaction between the two factors was significant, *F*(3, 232) = 4.350, *MSE* = .019, *p* = .005, $\eta_{p}^{2}=.053$, indicating that although developmental improvement was evident across the two experiments, Year 1 children were less accurate in Experiment 1B than 1A, *F*(1, 59) = 5.030, *MSE* = .019, *p* = .029, $\eta_{p}^{2}=.079$, while the adults were more accurate in Experiment 1B than 1A, *F*(1, 62) = 9.187, *MSE* = .016, *p* = .004, $\eta_{p}^{2}=.129$.

Analysis of the preparatory interval data led to the following exclusions: one child from Year 1 was excluded as they did not manage to recall any items and two children from Year 3 due to a fault in the data output from the programme. While the age effect was still apparent, *F*(3, 229) = 25.845, *MSE* = 2.677, *p* < .001, $\eta_{p}^{2}=.253$, the two data sets also differed from each other, *F*(1, 229) = 18.761, *MSE* = 2.677, *p* < .001, $\eta_{p}^{2}=.076$. Participants in Experiment 1B performed significantly quicker overall than the participants in Experiment 1A (Experiment 1A: *M* = 3.839, *SE* = .122; Experiment 1B: *M* = 2.745, *SE* = .221). The interaction between the two factors, *F*(3, 229) = 14.500, *MSE* = 2.677, *p* < .001, $\eta_{p}^{2}=.160$, revealed that children in Years 1 and 3 responded quicker in Experiment 1B, *F*(1, 58) = 22.136, *MSE* = 6.280, *p* < .001, $\eta_{p}^{2}=.276$ and *F*(1, 59) = 8.333, *MSE* = 2.725, *p* = .005, $\eta_{p}^{2}=.124$. While Year 5 did not show a systematic difference across experiments, *F*(1, 50) = 1.039, *MSE* = 1.182, *p* = .313, $\eta_{p}^{2}=.020$, adults produced a marginal effect, revealing quicker intervals in Experiment 1B, *F*(1, 62) = 3.912, *MSE* = .465, *p* = .052, $\eta_{p}^{2}=.059$.

### Discussion

The aim of Experiment 1B was to confirm whether it was the external contextual cues, or the additional recall opportunity, that explained the increase in recalled items observed in Experiment 1A. In essence, neither children or adults were able to enhance their recall substantially merely with an additional recall opportunity. We did not observe a developmental change in regeneration impact, and the extent to which their total capacity increased was minimal in comparison to the cued conditions reported in Experiment 1A. Without the external support, memory representations are in an unrecoverable state that cannot be overcome with their own internal, strategic retrieval processes.

The delayed cued recall task provided similar developmental patterns in children’s and adults’ recall performance; across the two experiments, however, there were subtle differences between age groups. In the current experiment, the youngest 5- to 6-year-olds struggled to retrieve 25% of target items, which was less than Experiment 1A. But, from the age of 8 years, children’s ability to use the external, semantic cues to access the correct information increased into adulthood. The preparatory interval, the gap between the end of the cue administration, and the participants’ response also changed across the two experiments. While 5- to 8-year-old children responded significantly quicker in Experiment 1B, 9- to 10-year-olds were comparable on each task. A plausible explanation for this was a trade-off between accuracy and response delay. Correlations for each age group separately confirmed a negative trend between these two dependent measures, supporting this idea, albeit they were all non-significant, potentially due to the small sample sizes.

We conclude that cued listening span task facilitates recall because of the *cue* potency. The opportunity for regeneration does not explain the recall gain on its own, and it is evident that adults and children do not spontaneously regenerate memoranda that have not been actively maintained. One reason may be the challenge of generating their own internal cues to access target items in the working memory system. The final step is to take our understanding of these tasks further and understand the relationships between our children’s measures of SM to explain individual differences in adults’ and children’s WMC.

## Individual differences

Here, we investigate the key inter-relationships between performance measures across all tasks. Our objective is to identify whether our novel estimates of SM in stressing search and retrieval processes can account for variance in WMC.

We derived z-scores for all variables within each age group and used these in the subsequent analyses. We reverse-scored the response time data to simplify the interpretation (multiplying each *z* score × −1) such that higher scores represented better performance and are referred to as “recall rate” in this case. A composite measure for the delayed cued recall task was also generated by averaging the two *z*-scores. Participants’ WMC score was derived from participants’ baseline WMC taken from the cued listening span task in Experiments 1A and 1B. The three children who were excluded from the RT analyses in Experiment 1B were not included in any subsequent analyses due to incomplete data sets.

We examined three sets of hypotheses. First, we asked whether our SM estimates were linked to the baseline WMC, drawing from all participants in Experiments 1A and 1B. We found moderate to strong zero-order correlations between all measures (Table 3). Thus, individuals with a higher WMC recalled more items on the delayed cued recall task and did so more quickly. These correlations remained significant even when partialling our age and experiment version, although delayed cued recall accuracy and rate were no longer associated. When each experiment was analysed separately, partialling out age, neither experiments showed this correlation to be significant, Experiment 1A: *r*(172) = .008, *p* = .913; Experiment 1B: *r*(54) = .133, *p* = .329.

**Table 3.** The inter-correlations between the delayed cued recall measures and WMC.

	1	2	3	4
1. Delayed recall rate	–	.412***	.842***	.702***
2. Delayed recall accuracy	−.047	–	.838***	.597***
3. Delayed composite	.692***	.689***	–	.773***
4. WMC	.371***	.161*	.385***	–

* *p* < .05; ** *p* < .01; *** *p* < .001

WMC: working memory capacity.

Top triangle: zero-order correlations; bottom triangle: partial correlations controlling for age and experiment version.

Second, we examined variance in recall gain, the benefit from having recall cues for listening span, alongside the delayed recall performance (see Table 4). At this point, we could only use participants from the cued listening span conditions in Experiment 1A.^6^ We found moderate to strong zero-order correlations between recall gain from the cued listening span task, delayed cued recall, and WMC. Partialling out cue condition did not materially change this pattern. In other words, those who could utilise cues to reconstruct previously inaccessible memoranda were faster and more accurate at responding in delayed cued recall and exhibited a larger WMC.

**Table 4.** The inter-correlations between recall gain from the cued listening span task, the delayed cued recall measures, and WMC.

	Delayed cued recall rate	Delayed cued recall accuracy	Delayed cued recall composite	WMC
Recall gain (zero-order)	.565***	.496***	.616***	.781***
Recall gain (condition partialled)	.585***	.486***	.621***	.792***
Recall gain (age partialled)	.136	.077	.151	.554***

* p < .05; ** p < .01; *** p < .001

WMC: working memory capacity.

The correlation between recall gain and WMC remained significant after partialling out age. However, the correlations between recall gain and delayed cued recall tasks were no longer reliable, implying age acts as a mediating factor. Assessing adult and children samples separately, the adult data generated correlations close to zero between the delayed cued recall and recall gain, recall rate: *r*(32) = .067, *p* = .714; accuracy: *r*(32) = −.094, *p* = .608; composite: *r*(32) = −.072, *p* = .694. Therefore, there was very little linking the external support provided by the cued listening span task and the delayed cued recall. However, children did exhibit zero-order correlations between these variables; recall gain significantly correlated with the three delayed cued recall measures, recall rate: *r*(83) = .391, *p* < .001; accuracy: *r*(83) = .336, *p* = .002; composite: *r*(83) = .454, *p* < .001.

Third, we asked whether variance in WMC can be attributed to search and retrieval processes, that is, estimates of SM. The correlation between age and WMC was strong. Therefore, a defining test of this hypothesis is whether a SM construct can substantially reduce the potency of WMC–age associations. As shown in the summary in Table 5, partialling out both SM measures leaves the age and WMC relationship non-significant. This is consistent with the argument that the SM construct may be an important component of the variance in this measure of WMC in adults and children.

**Table 5.** The breakdown of the age–WMC correlation, partialling recall gain from the cued listening span task and the delayed cued recall composite measures.

	Correlation age–WMC
Zero-order correlation	.775 (*p* < .001)
Partialling out recall gain from cued listening span	.540 (*p* < .001)
Partialling out delayed cued recall composite	.349 (*p* < .001)
Partialling out recall gain and delayed cued recall	.167 (*p* = .077)

WMC: working memory capacity.

To investigate this further, we ran a series of linear regression models—Table 6 reports the different steps and significance values. These examined the extent to which the two delayed cued recall measures and recall gain from the cued listening span task could explain the total variance surrounding participants’ baseline WMC. Age and condition were entered in the first step of each regression to control for any general age- and condition-related effects. Next, the delayed cued recall or recall gain measures were entered as either the second or third steps. The final step examined whether our three measures could account for significant, unique variance of WMC, above and beyond the previously entered measures (underlined in Table 6). The final model accounted for 80.3% of the total variance, *F*(5, 109) = 88.813, *p* < .001. Age and condition in the first step explained the majority (60.1%) of the variance. The remaining 20.2% was systematically linked with the three different measures, each explaining their own significant, unique contribution to individual differences in WMC.

**Table 6.** Hierarchical regressions predicting variation in WMC.

Step	IV	*R²*	Δ*R²*	*F*	*df*	*p*
1	Age and condition	.601	.601	84.506	2,112	.001
2	Delayed cued recall accuracy and recall gain	.735	.134	27.814	2,110	.001
3	Delayed cued recall rate	.803	.068	37.396	1,109	.001
2	Delayed cued recall rate and recall gain	.795	.194	52.175	2,110	.001
3	Delayed cued recall accuracy	.803	.007	4.120	1,109	.045
2	Delayed cued recall rate and accuracy	.708	.107	20.163	2,110	.001
3	Recall gain	.803	.095	52.299	1,109	.001

WMC: working memory capacity.

**Figure 6.**
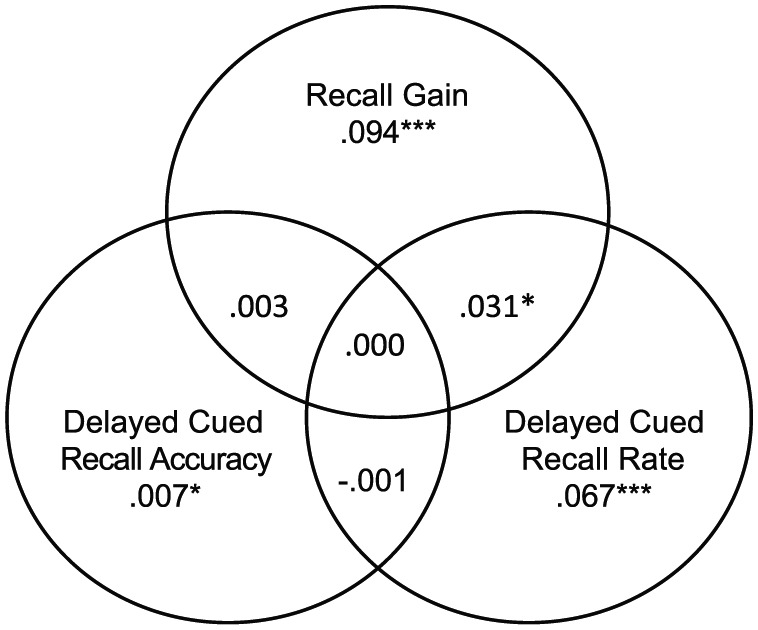
Venn diagrams representing the shared and unique variance accounted for by recall gain taken from the cued listening span task and accuracy and recall rate from the delayed cued recall task. Numbers are based on the regressions reported in Table 6. * *p* < .05; ** *p* < .01; *** *p* < .001

Finally, variance partitioning was undertaken to identify how the 20.2% of variance could be attributed among the three different SM measures—recall gain in listening span, delayed cued recall accuracy, and recall rate (Figure 6, see Hall, Jarrold, Towse, & Zarandi, 2015; Salthouse, 1994). All three measures accounted for unique contributions to WMC, and the largest contribution was made by recall gain, that is, the ability to use the external cues to identify previously inaccessible memoranda. Most shared variance was linked to recall gain and recall rate. Delayed cued recall accuracy shared minimal variance with other measures.

## Discussion

We have already demonstrated in Experiment 1A that children and adults can benefit from cues that potentially assist in reviving the retrieval of TBR information. Cues that reactivate sentential or semantic information about the sentence, and cues that are linked to the target words, can both facilitate accurate reporting of the target material. These data are fully consistent with the notion that reconstructive processes can occur in reading and listening span tasks (Towse et al., 2010), from as young as 5 years, and they are compatible with the ideas that SM processes of cued search are relevant to understanding WMC (Unsworth & Engle, 2007). Individual differences analysis further demonstrates that more direct estimates of SM processes from the impact of cues in listening span and delayed recall are relevant to understanding variance in WMC itself. Recently, Loaiza and Camos (2018) provided evidence that semantic cues benefit working memory recall among adults. While they addressed a different set of questions (e.g., the contrast between semantic and phonological support, the difference between semantic support and rehearsal), their data converge with ours in drawing attention to meaning-based support for recall.

When completing the delayed cued recall task, the time taken for participants to search and access the target memory representations (preparatory interval) and their recall of the representation was positively correlated with WMC. Convergent with these results, recall gain, that is, participants’ ability to benefit from the external support on the listening span task, also correlated with WMC. Individuals who could utilise the reconstructive benefit of the external contextual support to reactivate and retrieve select memoranda successfully were linked to a larger WMC.

Unsurprisingly age is a major contributor to variance in WMC in our data. This provided an opportunity to test whether there was evidence that SM estimates could explain this link. Indeed, controlling for recall gain and delayed cued recall substantially attenuated the age–WMC association. The ability to use retrieval cues to reconstruct and retrieve memoranda from long-term memory as a mediating factor between age and WMC converges with Miyake and Friedman (2004) of which our delayed cued recall task was based upon. They reported that the link between reading span performance and cognitive ability was rendered non-significant after partialling out delayed cued recall performance.

In the present data, regression analyses, including variance partitioning, showed that all three SM measures contributed significantly to the model, with the largest amount of shared variance between recall gain and delayed cued recall rate. Somewhat to our surprise, delayed cued recall accuracy did not contribute in the same way. We do not have a full account of why this is so, but we stress that recall accuracy was not always high, and therefore, guessing/mediational deficiencies may obscure the predictive impact of this variant with the current sample.

## General discussion

Our data offer an important developmental perspective on contextual, cue-dependent search processes in WMC, otherwise referred to as SM (Unsworth & Engle, 2007). Several theoretical accounts of working memory propose that only a small subset of information can be actively maintained, while other memory representations are held in long-term memory (Healey & Miyake, 2009; Unsworth & Engle, 2007) or outside the focus of attention (Cowan, 1999), which may help scaffold or support specific memories (Towse et al., 2010). A common assumption is that in a complex span task, a non-recalled item is forgotten. Yet this is evidently not always the case. The current analyses show this to be an oversimplification—children from the age of 5 years and adults can recover fragile memory representations with a variety of cues or prompts. We also demonstrate that some cues may be more effective than others.

Children’s immediate memory—sometimes termed PM—is small, on average two items at the age of 5 years (Hall et al., 2015; Jarrold et al., 2015; Roome et al., 2014). PM serves to maintain a specific number of representations under the allocation of attention. Once attention is captured elsewhere, they may become rapidly inaccessible in PM. SM seems to follow a protracted development, across typical (Roome et al., 2014) and atypical (Gibson, Gondoli, Flies, Dobrzenski, & Unsworth, 2010) populations. The current data offer potentially important insights as to *why* children find it so difficult to use SM. Children may find it difficult to take advantage of categorical cues or spontaneously generate effective internal search strategies to reconstruct less accessible representations within working memory (Healey & Miyake, 2009; Towse et al., 2010; Unsworth & Engle, 2007). Cued recall is a demanding environment for children to report memoranda despite the external contextual support. Importantly though, we have shown that other forms of (more associative) support can boost their performance substantially (with respect to effect sizes and absolute recall levels).

We readily acknowledge several limitations in this study. First, while the overall sample size was reasonably large for a study of this type, and the effect sizes of cue impact in listening span were very large, sampling for individual conditions in each age group were modest. Accordingly, we emphasise the main effects that have been found rather than the detail of age group changes. Second, we did not implement an independent measure or metric of baseline WMC. This would have allowed for a more complete test of how SM is linked to WMC, by deriving a broader statistical construct on the latter. Third, we limited our remit here to evaluating semantic reconstruction of verbal memoranda. Insofar as retrieval from SM is a domain-general memory system, and recognises multiple sources of reconstructive support, additional analysis with a variety of verbal and non-verbal stimuli would be informative, especially in light of claims for sudden and complete loss of visual information in other paradigms (Zhang & Luck, 2009).

Overall, we provide convergent evidence that working memory representations are not held in an all-or-nothing state. Moreover, we provide evidence that recall processes are importantly intricate and relevant to working memory (Miyake & Friedman, 2004), more so than some accounts of the development of WMC have realised. The data support the view that working memory representations can be fragile and partial, but they can also be supported and their recall facilitated, as predicted by the recall reconstruction hypothesis (Towse et al., 2010). Children as young as 5 years of age are able to draw upon cues to the target memoranda in listening span. Even more so, children benefit from cues that remind children about the processing sentence in listening span that led to the target memoranda. Reconstructive processes facilitate the production of representations and shape individual differences through different cues that operate through partially independent mechanisms within the working memory system.

## Supplemental Material

QJE-STD_16-335R3-Supplemental_Material – Supplemental material for Contextual support for children’s recall within working memory Click here for additional data file. Supplemental material, QJE-STD_16-335.R3-Supplemental_Material for Contextual support for children’s recall within working memory by Hannah E Roome, John N Towse and Maria M Crespo-Llado in Quarterly Journal of Experimental Psychology

## Notes

1. The programming of all tasks is available upon request to the corresponding author.

2. Adults and children were given different distractor activities to occupy their attention in the verbal domain, while it not being so difficult that the activity caused undue fatigue (children) or being so easy that they were able to actively maintain the memoranda (adults).

3. Using Bonferroni pairwise comparisons here and for all subsequent post hoc analyses.

4. The cue benefit was calculated using the total number of cues that successfully triggered the retrieval of TBR items, divided by the total number of cues administered. Of course, the number of cues administered was the inverse of the baseline performance, since we did not cue already remembered items.

5. We appreciate the constructive recommendation to implement an additional control condition for the cued listening span task made during the review process. Consequently, we undertook additional data collection, reported in Experiment 1B.

6. Recall gain was calculated as the difference in the proportion of total correct recall before and after cue administration. The cue gain scores were also reverse-scored and converted to *z*-scores.
