# Supplementary material for: Contextual support for children’s recall within working memory
Source: Q J Exp Psychol (Hove). 2018 Oct 24;72(6):1364–78. doi: 10.1177/1747021818804440 (PMC6535801; doi:10.1177/1747021818804440)
Supplement: QJE-STD_16-335R3-Supplemental_Material – Supplemental material for Contextual support for children’s recall within working memory [file QJE-STD_16-335R3-Supplemental_Material.docx]

Supplementary Material for

# Contextual Support for Children’s Recall within Working Memory

Hannah E. Roome, John N. Towse and Maria M. Crespo-Llado

Supplementary Material 1: The sentences used in the cued listening span task, and the sentence- and word-cues used in the cued conditions.

| **Sentence** | **Target** | **Sentence-cue** | **Word-cue** | **Length of recording** | **Words/second** |
| --- | --- | --- | --- | --- | --- |
| I like to eat fish and | Chips | Fish | Potatoes | 1.752s | 3.425 |
| A baker made a loaf of | Bread | Baker | Sandwich | 1.664s | 3.606 |
| I can see with my | Eyes | See | Face | 1.515s | 3.300 |
| A spider has eight | Legs | Spider | Arms | 1.655s | 2.417 |
| A house is made of | Bricks | House | Wall | 1.716s | 2.914 |
| I am tired at the end of the | Day | Tired | Week | 1.806s | 4.430 |
| I can hear with my | Ears | Hear | Music | 1.504s | 3.324 |
| The number after two is | Three | Number | Triplet | 1.884s | 2.654 |
| I sat down on a | Chair | Sat | Table | 1.545s | 3.236 |
| A pig has a curly | Tail | Pig | Wagging | 1.513s | 3.305 |
| I wear socks on my | Feet | Socks | Toes | 1.597s | 3.131 |
| A giraffe has a long | Neck | Giraffe | Scarf | 1.680s | 2.976 |
| A postbox is coloured | Red | Postbox | Danger | 1.814s | 2.205 |
| I waved goodbye with my | Hands | Wave | Gloves | 1.816s | 2.753 |
| I wear a hat on my | Head | Hat | Body | 1.912s | 3.138 |
| The sun is in the | Sky | Sun | Blue | 1.524s | 3.281 |
| I sweep the floor with a | Broom | Witch | Sweep | 1.567s | 3.829 |
| Birds have wings to | Fly | Birds | Airplane | 1.467s | 2.727 |
| I eat my dinner with a knife and | Fork | Dinner | Spoon | 1.902s | 4.206 |
| Ice cream is very | Cold | Ice cream | Hot/Shiver | 1.349s | 2.965 |
| Sharks swim in the | Sea | Sharks | Water | 1.551s | 2.579 |
| A farmer lives on a | Farm | Farmer | Tractor | 1.578s | 3.169 |
| If it rains I will get | Wet | Rain | Dry | 1.909s | 3.143 |
| Rockets fly in outer | Space | Rockets | Planets | 1.719s | 2.327 |
| The magician waved his magic | Wand | Magician | Fairy | 1.834s | 2.726 |
| I use a toothbrush to clean my | Teeth | Toothbrush | Mouth | 2.012s | 3.479 |
| When I am thirsty I have a | Drink | Thirsty | Juice | 2.086s | 3.356 |
| You can see the moon at | Night | Moon | Day | 1.764s | 3.401 |
| The football team scored a | Goal | Football | Net | 1.842s | 2.714 |
| To make a snowman you need | Snow | Snowman | Flakes | 1.81s | 3.315 |
| Worms live under | Ground | Worms | Gardening | 1.325s | 2.264 |
| A fireman helped put out the | Fire | Fireman | Hot | 1.625s | 3.692 |
| I skip with a skipping | Rope | Skip | Knot | 1.709s | 2.926 |
| Wool comes from | Sheep | Wool | Farm | 1.340s | 2.239 |
| A joke makes me | Laugh | Joke | Tickle | 1.472s | 2.717 |
| I go to the library to read a | Book | Library | Story | 2.114s | 3.784 |
| I washed the dirty dog until it was | Clean | Dog | Soap | 2.432s | 3.289 |
| I get hungry if I have no | Food | Hungry | Dinner | 1.894s | 3.696 |
| Leaves grow on big | Trees | Leaves | Forest | 1.637s | 2.443 |
| I crossed the river in a rowing | Boat | River | Ship | 1.730s | 4.046 |
| Leaves blow in the | Wind | Leaves | Weather | 1.637s | 2.443 |
| Everyday I comb my | Hair | Comb | Curls | 1.775s | 2.254 |
| Before I go to school I eat my | Breakfast | School | Cereal | - | - |
| A baby cat is called a | Kitten | Cat | Baby | - | - |

Supplementary Material 2: The stimulus pool for the delayed cued recall task based on Morrison, Chappell and Ellis (1997)

|  | **Category** | **Stimuli** | **Objective AoA (75%) (months)** |
| --- | --- | --- | --- |
| 1 | Animal | Zebra | 44.5 |
| 2 | Animal | Camel | 68.5 |
| 3 | Animal | Monkey | 25.1 |
| 4 | Animal | Donkey | 50.5 |
| 5 | Insect | Butterfly | 23.4 |
| 6 | Insect | Ladybird | 38.5 |
| 7 | Insect | Spider | 25.1 |
| 8 | Insect | Bee | 56.5 |
| 9 | Transport | Airplane | 23.4 |
| 10 | Transport | Motorbike | 38.5 |
| 11 | Transport | Lorry | 44.5 |
| 12 | Transport | Van | 50.5 |
| 13 | Tool | Hammer | 25.1 |
| 14 | Tool | Nail | 68.5 |
| 15 | Tool | Screwdriver | 68.5 |
| 16 | Clothing | Jacket | 56.5 |
| 17 | Clothing | Sock | 23.4 |
| 18 | Clothing | Skirt | 56.5 |
| 19 | Body Part | Eye | 44.5 |
| 20 | Body Part | Hand | 23.4 |
| 21 | Body Part | Nose | 56.5 |
| 22 | Body Part | Thumb | 38.5 |
| 23 | Fruit | Cherry | 44.5 |
| 24 | Fruit | Grapes | 74.5 |
| 25 | Fruit | Pear | 56.5 |
| 26 | Vegetable | Mushroom | 62.5 |
| 27 | Vegetable | Onion | 68.5 |
| 28 | Vegetable | Lettuce | 74.5 |
| 29 | Fictional Character | Wizard | 56.5 |
| 30 | Fictional Character | Mermaid | 50.5 |
| 31 | Fictional Character | Fairy | 62.5 |
| 32 | Fictional Character | Witch | 50.5 |
| 33 | Musical Instrument | Trumpet | 56.5 |
| 34 | Musical Instrument | Violin | 62.5 |
| 35 | Musical Instrument | Drum | 50.5 |
| 37 | Musical Instrument | Guitar | 62.5 |
| 38 | Bird | Penguin | 38.5 |
| 39 | Bird | Swan | 62.5 |
| 40 | Bird | Owl | 38.5 |
| 41 | Household item | Iron | 44.5 |
| 42 | Household Item | Telephone | 23.4 |
| 43 | Household Item | Lamp | 74.5 |
| 44 | Household Item | Bath | 23.4 |
| 45 | Stationary | Pencil | 38.5 |
| 46 | Stationary | Ruler | 62.5 |
| 47 | Stationary | Scissors | 23.4 |
| 48 | Job | Nurse | 50.5 |
| 49 | Job | Doctor | 44.5 |
| 50 | Weapon | Gun | 44.5 |
| 51 | Weapon | Sword | 50.5 |
